# Representation of rewards differing in their hedonic valence in the caudate nucleus correlates with the performance in a problem-solving task in dogs (*Canis familiaris*)

**DOI:** 10.1038/s41598-023-40539-1

**Published:** 2023-09-01

**Authors:** Laura V. Cuaya, Raúl Hernández-Pérez, Attila Andics, Rita Báji, Márta Gácsi, Marion Guilloux, Alice Roche, Laurence Callejon, Ádám Miklósi, Dorottya Júlia Ujfalussy

**Affiliations:** 1 Department of Ethology, Institute of Biology, Eötvös Loránd University, Budapest, Hungary; 2 MTA-ELTE ‘Lendület’ Neuroethology of Communication Research Group, Hungarian Academy of Sciences – Eötvös Loránd University, Budapest, Hungary; 3 ELTE NAP Canine Brain Research Group, Budapest, Hungary; 4 ELKH-ELTE Comparative Ethology Research Group, Budapest, Hungary; 5 Symrise Pet Food - Spécialités Pet Food SAS, Elven, France; 6 Psychobiology Research Group - NAP, Institute of Cognitive Neuroscience and Psychology, Research Centre for Natural Sciences, Budapest, Hungary; 7 MTA-ELTE Lendület “Momentum” Companion Animal Research Group, Budapest, Hungary; 8 Department of Ethology, ELTE Eötvös Loránd University, Pázmány Péter sétány 1/C, Budapest, 1117 Hungary

**Keywords:** Neuroscience, Psychology

## Abstract

We have investigated dogs’ (*Canis familiaris*) abilities in associating different sounds with appetitive stimuli of different incentive values. The association’s establishment was first tested on family dogs (n = 20) in a problem-solving behavioural paradigm (experiment 1), then in a problem-solving behavioural paradigm as well as an fMRI study on specially trained family dogs (n = 20) (experiment 2). The aim was to show behavioural and parallel neural effects of the association formed between the two sounds and two different associated appetitive stimuli. The latency of solving the problem was considered an indicator of the motivational state. In our first experiment, where only behaviour was studied, we found that dogs were quicker in solving a problem upon hearing the sound associated with food higher in reward value, suggesting that they have successfully associated the sounds with the corresponding food value. In our second experiment, this behaviour difference was not significant. In the fMRI study, the cerebral response to the two sounds was compared both before and after the associative training. Two bilateral regions of interest were explored: the caudate nucleus and the amygdala. After the associative training, the response in the caudate nucleus was higher to the sound related to a higher reward value food than to the sound related to a lower reward value food, which difference was not present before the associative training. We found an increase in the amygdala response to both sounds after the training. In a whole-brain representational similarity analysis, we found that cerebral patterns in the caudate nucleus to the two sounds were different only after the training. Moreover, we found a positive correlation between the dissimilarity index in the caudate nucleus for activation responses to the two sounds and the difference in latencies (i.e. high reward value associated sound condition latency—low reward value associated sound condition latency) to solve the behavioural task: the bigger the difference between the conditions in latency to solve the task, the greater the difference in the neural representation of the two sounds was. In summary, family dogs’ brain activation patterns reflected their expectations based on what they learned about the relationship between two sounds and their associated appetitive stimuli.

## Introduction

Animals are regularly confronted with different types of rewards that elicit attention and approach of various intensities, some of which are associated with distinct acoustic cues. Members of the family *Canidae* can predict the presence of specific prey type upon hearing specific sounds^1^, while dogs readily learn to perform auditory recognition tasks under laboratory settings^2–4^.

Secondary reinforcers, such as associated specific sound stimuli, have been found to provoke similar neural activation patterns as the primary reinforcers in humans^5^ as well as in rats^6^. In humans, reward-sensitive brain regions are also activated during reward anticipation^7^. Moreover, the degree of activation covaries with motivational value in reward-sensitive regions: highly motivational stimuli promote a stronger activation in reward-sensitive regions than less motivational stimuli^8^.

There is convincing evidence that animals show preference both for higher quantity and better quality reward e.g.^9,10^. Reward quality dependent anticipation has been demonstrated in rats using single unit recordings^11^, results suggesting nucleus accumbens neurons respond not only to reward but also to reward anticipation while also representing difference in reward quality.

In the case of humans, the striatum and the amygdala have a complementary role in the neural representation of associative reward learning (i.e. the ability to detect a contingency between a stimulus and a positive outcome)^12,13^. The striatum processes the reward value related to conditioned stimuli, whereas the amygdala enhances the learning process by updating the associations and promoting attention to salient stimuli^5,14,15^.

Neuroimaging studies in dogs have revealed the central role of the caudate nucleus (CN) and the amygdala in associative reward learning. Two studies showed an increase in activity in the CN related to a hand signal that predicted a food reward in comparison to a hand signal that predicted no reward^16–18^ and similar results were obtained using a predictive outcome praise^19^. Another study^20^, reported that among five scents (familiar human, strange human, familiar dog, strange dog, self), the maximum activation in the dogs’ CN was found in response to the scent of a familiar human, suggesting a positive association between the scent and the human. As human scent, especially that of a familiar human may be considered a reward in itself, this study, in a sense similar to ours, contrasts the neural response to rewards differing in quality. Another study found that both, the CN and the amygdala increased their response to the conditioned stimuli related to a positive outcome in comparison with the neutral one, independent of the sensory modality (visual, olfactory or hearing). The authors suggested that similarly to humans, the CN in dogs is related to reward representation, and the amygdala is related to signalling stimulus salience^21^. A subsequent olfactory study revealed that the amygdala exhibits a greater response to an odour associated with a reward compared to an odour associated with nothing and a novel odour^22^. When exploring human speech processing by dogs, increased activity in primary reward regions (CN and ventral striatum) was found when both lexical and intonational information were consistent with praise^23^. CN also increased its response to human faces, especially to human faces expressing happiness^24,25^. Taken together, these studies highlight the role of the CN in associative reward learning across different predictive outcomes (i.e. food, praise, familiar human scent and human faces) and across sensory modalities.

Most of the studies on dogs have directly compared *reward expectation* vs. *no reward expectation*, while in their everyday life dogs are regularly confronted with different types of rewards and not only the presence or absence of the reward. It has been shown that dogs can distinguish food qualitatively, for example, inequity aversion studies have suggested that dogs discriminate between two types of food differing in value^26^, and in a delay gratification paradigm dogs have been shown to be willing to wait for high-value food^27^.

Here, an associative learning paradigm was used to explore behavioural motivational changes related to the expectation of an appetitive stimulus (from now on we use the phrase “reward”), to study their connections and relationship with the neuronal representation of expectations of two different quality rewards in the dog brain through functional magnetic resonance imaging (fMRI). Classical conditioning was used to teach dogs an association of two tones with two types of food respectively (high reward value (HRV) food: meat treats and low reward value (LRV) food: low-sugar biscuits). Next, it was tested whether hearing one of the two conditioned stimuli has a differential impact on the latency of solving an identical task (opening a parcel to get the food reward). To detect the brain response evoked by both tones (related to the two types of food) in two regions of interest (the CN and the amygdala), we presented awake, trained dogs with the two tones before and after classical conditioning, while we acquired functional images of their brains. At the behavioural level, we hypothesized that after the association training dogs will open the parcel faster hearing the sound associated with the HRV food, compared to hearing the sound associated with the LRV food. At the cerebral level, we hypothesized that after training the CN will show a higher activity related to the sound associated with HRV food. We also predicted increased activity in the amygdala related to learning, with activation being more pronounced in this region after association training than before.

In our first experiment, we aimed to see if dogs can associate two different tones to two types of food differing in reward value, and test if this association formation manifests in a different motivational state in problem-solving. Subsequently, in our second experiment, we wanted to check if this difference in the motivational state is mirrored in the brain response, thus repeated the first experiment using fMRI-trained subjects.

## Experiment 1

In the first experiment, we tested whether hearing sounds predicting better food quality resulted in faster problem-solving (removing a standard three-layer wrapping of a parcel) in relation to hearing sounds associated with less preferred food in family dogs. We trained subjects to associate two specific sounds to corresponding foods, one of low and one of high quality.

### Method

#### Subjects

Twenty adult family dogs (eleven males, nine females; age range 1 to 10 years old, mean age = 4.7, SD = 2.9) of various breeds (four Hungarian vizslas, four mixed breeds, three Golden Retriever, two Jack Russell terriers, one Chihuahua, one Airedale terrier, one Groenendael, one Boxer, one Parson Russell terrier, one Australian shepherd, one Springer spaniel). Owners were asked about their dogs’ preferences regarding the two types of appetitive stimuli used (cooked ham and unsweetened fibre cookies). This information has been noted connected to subject ID. A clear preference for cooked ham was reported for all subjects. All dogs enrolled in pre-training achieved pre-training criteria and completed the study. All experimental procedures were approved by the National Animal Experimentation Ethics Committee (number of ethical permission: PE/EA/1505-5/2017). All owners signed an informed consent form. Dogs and owners could leave the sessions at any time. Detailed subject data may be found in the Supplementary Material.

#### Stimuli

Sound stimuli were created using the Adobe® Audition software. The frequency of the low-pitched tone was 120 Hz, while that of the high-pitched tone was 220 Hz, both well within the hearing range of both dogs (subjects) and humans (experimenters). Dogs’ hearing range covers that of humans (64–23 000 Hz), however, exceeds that considerably in the high-frequency domain ranging approximately from 67- 45 000 Hz^28^. The sound intensity was set at 60 ± 10 dB. At this intensity, both used frequencies are well detectable for dogs^29^. The 100 Hz difference makes the two tones clearly distinguishable.

#### Procedure

The behavioural study was comprised of three phases: pre-training on the problem-solving task, associative conditioning, and testing. All sessions were video recorded.

##### Pre-training and baseline testing

**Figure 1:**
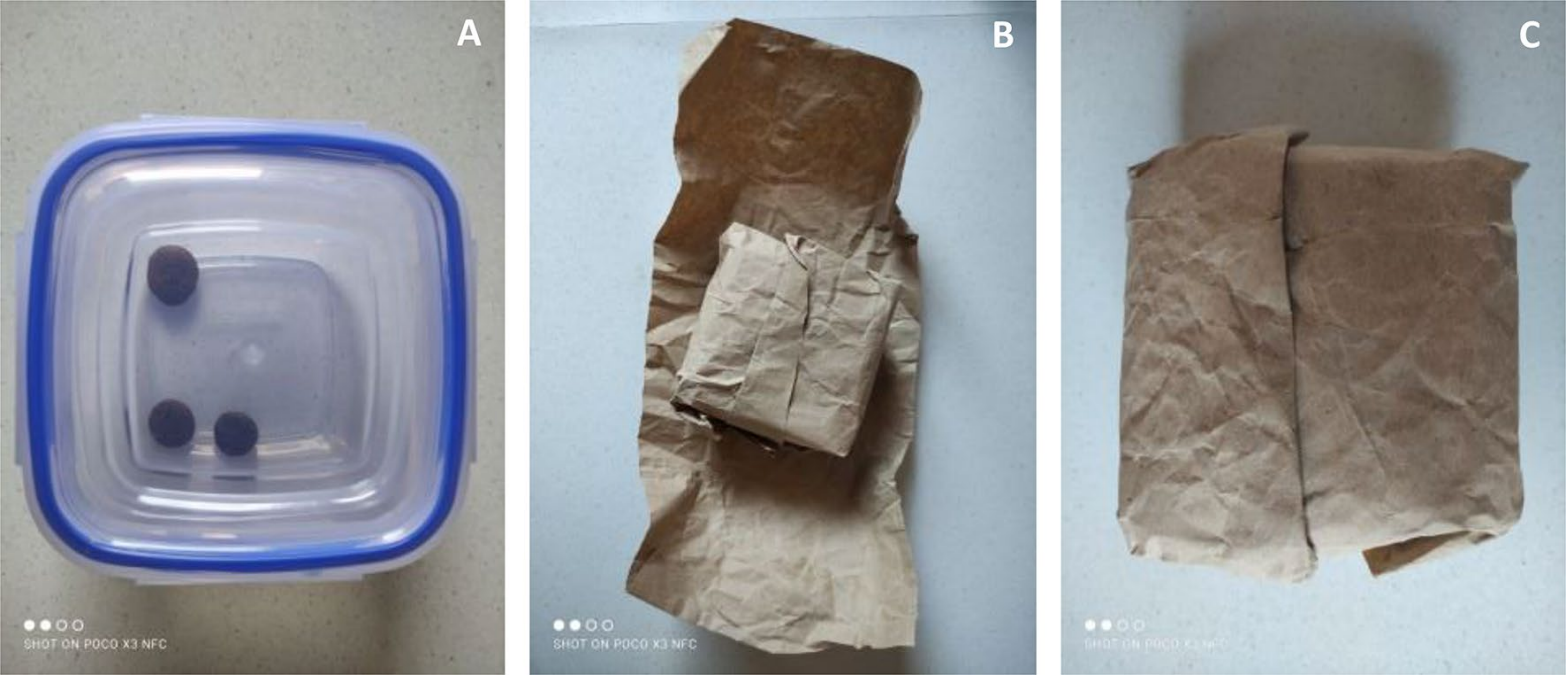
Illustration of the parcel and its wrapping – (**A**) Unwrapped box. (**B**) Box partially wrapped. (**C**) Standardly wrapped box in three layers.

Dogs were trained at home by their owner to open a parcel (standardly packed in three layers of brown paper, secured with a single 5 cm transparent tape – see Fig. 1) until dogs became capable to open the parcel without looking at their owner and without encouragement from the owner within three minutes. Parcel contained a plastic box baited with some pieces of the dog’s daily diet. Once the training was completed, the dogs’ performance in opening the parcel according to the criterion was tested by an experimenter at the Department of Ethology. The criterion was set as the dog being able to entirely strip the parcel of all three layers of brown paper independently within 180 s. This criterion testing was videotaped, coded and used as a baseline for data analysis.

##### Associative learning

Dogs received a sound signal followed by an appetitive stimulus (i.e. “food reward”) assigned to the sound signal. We used two different sounds (high-pitched and low-pitched) signalling two different foods (HRV, meat treats: cooked smoked ham; LRV, zero-sugar fibre cookies: Detki® Sugar-free Household Biscuits). The tone-food pairing was counterbalanced across participants, so the high-pitched tone was paired with HRV food for eleven dogs and was paired with the LRV food for nine dogs. Each session consisted of 24 consecutive training trials. Sounds were presented in a semi-random order, 12 high-pitched and 12 low-pitched, each for 1 s, with a 10-s break between sounds, when the adequate appetitive stimulus was administered and consumed.

Association training was performed on three occasions, on two training days and the testing day, directly before testing. The number of days between occasions was maximized at 5. On the first training day, parcel opening to criterion was videotaped, and then dogs participated in 3 training sessions with 5 min. breaks in between. During the first training session the orientation of subjects at the bowls containing the appetitive stimuli was noted to validate owner reported preference for cooked ham over unsweetened fibre cookies. A clear preference was noted in all subjects. On the second training day, following the 3 training sessions dogs were given the task to open a standard box with meat treats inside, while listening to their HRV-food associated sound (1 s), at 2 s intervals and a standard box with fibre cookies treats inside while listening to their LRV-food associated sound (1 s), played at 2 s intervals. This procedure was meant to tie the sounds and the parcel-opening task together. On the third training day (also the testing day), dogs participated in 2 training sessions with 5-min-long breaks in between. After a 10-min break dogs participated in the testing session.

##### Testing

The task was to open a parcel (wrapped box cleaned and empty to avoid olfactory cueing) with one of the previously conditioned tones displayed for a 1-s duration every 2 s. The test continued until the parcel was stripped of all three layers of wrapping paper or was terminated at three minutes. Reward adequate to the sound was supplied by the experimenter in a box similar to the one found inside the parcel, as soon as the parcel was successfully opened. This exchange of boxes (i.e. non-baited to baited) was intended not to weaken the association and was made least conspicuous as possible. After a 5-min delay, the task was repeated with the other tone. The order of the testing trials was counterbalanced across dogs. Testing trials were videotaped and coded and data was used as the basis of analyses.

#### Data analysis

Baseline and testing trials were coded for latencies to open the parcel. Latencies were calculated in seconds from the first touch of the parcel until all wrapping paper was removed from the plastic container. We calculated the difference in opening latencies by subtracting the latency to open the box related to the low reward value (HRV) food sound from the latency to open the box related to the high reward value (LRV) food sound. We calculated the difference in latencies per participant. Positive differences in latencies indicate that the dog opened the box related to the HRV food sound faster. In contrast, negative differences indicate that the dog opened the box related to the LRV food sound faster. A difference of zero indicated no difference in the dog’s latency to open either box. We have cross-coded 75% of video recordings and calculated overall interobserver agreements by Intraclass Correlation Coefficients (IBM SPSS Statistics 28.0.0.0) (average measure ICC = 0.975 with a 95% confidence interval from 0.958 to 0.985 (F(59, 58) = 40.698, p < 0.001). Raw data is provided as Supplementary Material. Performance of dogs in baseline, HRV-food and LRV-food conditions were compared. To compare opening latencies, we have built a Cox mixed-effects model fit by maximum likelihood.

### Results

**Figure 2:**
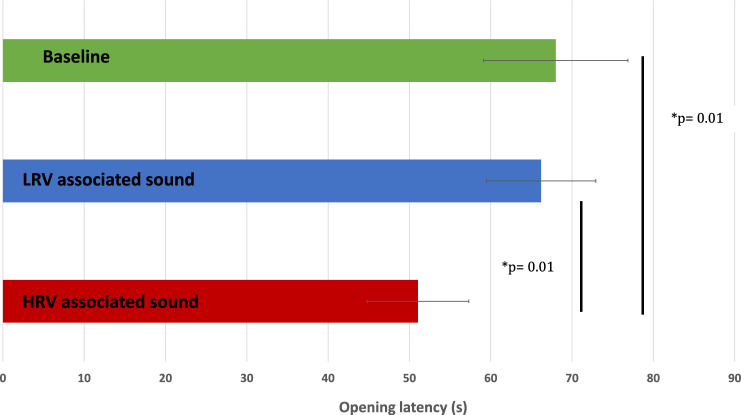
Group mean (± SE) of parcel opening latencies (s) in the three conditions (n = 20) in Experiment 1. Means and ranges(sec): Baseline: 68 (20–158); LRV condition: 66.2 (10–145); HRV condition: 51.05(14–96).

**Figure 3:**
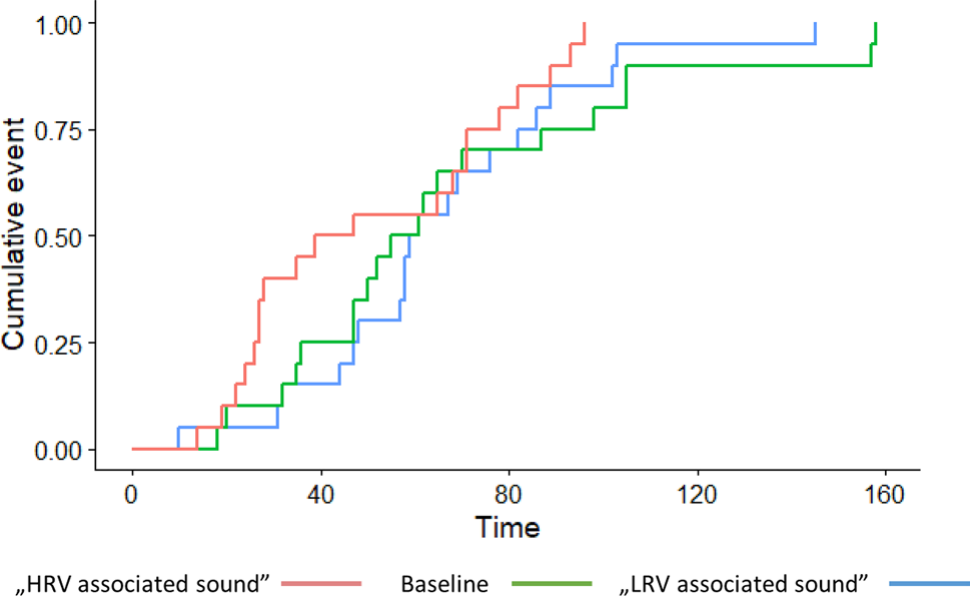
Parcel opening latencies (s) in the three conditions (n = 20).

Figure 2 shows the group opening latencies for the three conditions. Opening latencies were significantly lower when hearing the HRV-food-associated sound than when hearing the LRV-food-associated sound (p = 0.01) and from baseline (p = 0.01). The Cox mixed-effects model fit by maximum likelihood showed that the opening latencies, when hearing the LRV-food associated sound, did not differ significantly from baseline (exp (coef) = 0.908; p = 0.8). However, opening latencies, when hearing the HRV-food sound, were significantly lower than when hearing the LRV-food associated sound (exp (coef) = 2.66; p = 0.01) or baseline latencies (exp (coef) = 2.62; p = 0.01 (Fig. 3).

## Experiment 2

Based on the results of Experiment 1, we proceeded to run a second experiment with the aim of including both behavioural and fMRI tests on the same subject pool, fMRI-trained family dogs. This procedure would allow us to establish a connection between the dogs' behavioural responses and their brain representations of rewards. We did not use fMRI-trained dogs already in the first experiment, as training dogs to lie motionless in an fMRI scanner is a long process, requiring lots of joint efforts of dogs, trainers, and owners – so our trained subjects are a very precious resource. We only burden them and their owners with training and testing if we are confident that their efforts will not be in vain.

### Method

#### Subjects

Twenty adult family dogs (eleven females, nine males; age range 2 to 11 years old, mean age = 6 SD = 2.9) of various breeds, (one Australian shepherd, one Belgian shepherd, seven Border collies, one Labradoodle, two mixed breeds, three Cocker spaniels, one white Swiss shepherd, three Golden retrievers and one Springer spaniel) all trained to remain still inside an MRI scanner, participated (for the non-invasive measurement and training methods see^30^). None of these dogs participated in Experiment 1. Out of the 21 dogs enrolled in pre-training, 21 achieved pre-training criteria but only 20 completed the study. All experimental procedures were approved by the National Animal Experimentation Ethics Committee (number of ethical permission: PE/EA/1505-5/2017). Owners signed an informed consent form, and owners and dogs could leave the sessions at any time. Detailed subject data may be found in the Supplementary Material.

#### Stimuli

The same sound stimuli were used as in Experiment 1.

#### Procedure

##### Behaviour study

The same procedure as in Experiment 1, except an extra training day with three training sessions, was added to the training schedule. We increased the amount of training as some of the dogs in Experiment 1 did not show behaviour evidence (i.e. faster opening of the parcel upon hearing the sound associated with the higher reward value (HRV food) of forming the associations. The opening of baited parcels (see “Method” section of Experiment 1) has been moved to this extra training day. The only reason for this adjustment was to ensure a more robust sound-food association. Raw data is provided as Supplementary Material.

We have cross-coded 75% of video recordings and calculated overall interobserver agreements by Intraclass Correlation Coefficients (IBM SPSS Statistics 28.0.0.0) (average measure ICC = 0.975 with a 95% confidence interval from 0.958 to 0.985 (F(59, 58) = 40.698, p < 0.001).

##### fMRI study design

We used an event-related design, each event consisted of three repetitions of the same tone distributed over 7 s. Two event conditions were created: High-pitched tone and Low-pitched tone (same tones that were used in behaviour experiments). In each run, each type of event was repeated fifteen times and we also added 6 events of silence in a pseudo-random order (three events of the same condition were never presented consecutively). We create two unique runs. We acquired two runs before the behaviour experiment and two runs after the behaviour experiment. The post-training session was conducted within one to five days after completing the behaviour experiment. The post-training session started with special behavioural training, where the dogs were exposed to 24 trials, in which only 10 trials were followed by the reward. The partially rewarded training session aimed to familiarize dogs with sounds not being directly followed by reward and to minimize any potential frustration resulting from this experience in the scanner. This partially rewarded training session was in all other aspects similar to the previous behavioural training sessions. Then, we acquired the two post-training runs (in the inverse order of their pre-training runs).

#### Data acquisition

We used a sparse sampling acquisition in a 3 T Philips Ingenia scanner with an eight-channel dStream Paediatric Torso coil. Dogs were fitted with earmuffs to provide noise protection during scanning, and to present the stimuli. Blood-oxygen-level dependent (BOLD) images of the whole brain were acquired with a gradient-echo-echo-planar imaging (EPI) sequence (40 transverse slices, 2 mm thickness, 0.5 mm gap; TR = 10 s (1.680 s for acquisition); TE = 12 ms; flip angle = 90°; acquisition matrix 80 × 58; spatial resolution 2.5 mm × 2.5 mm; 37 volumes and 1 dummy scan). During the acquisition, the trainer and the owner remained inside the scanner room. We used as the exclusion criterion of movement, a maximum of 3 mm in any direction and less than 1° rotation in any direction (mean framewise displacement (FD)^31^) across all dogs and runs = 0.25 mm).

#### Data analysis

Image pre-processing and statistical analysis were performed using FSL^32^ version 5.0.11, and PyMVPA software package^33^.

##### Pre-processing

Each run was motion-corrected using MCFLIRT, spatially smoothed using a Gaussian kernel (FWHM 5 mm) and filtered using a high-pass filter to remove low-frequency signals.

For each dog, the acquired images were manually skull-stripped and spatially aligned to the first volume of the first run. We created a mean image by averaging all volumes of all runs. This mean image was then manually adjusted, using rigid-body and affine transformations, to match an anatomical template^34^, creating a normalized mean image, we calculated the transformation matrix from the mean functional to the normalized mean image and used it to transform each of the acquisitions to the anatomical template space.

##### Region of interest analysis

We used the General Linear Model (GLM) for the statistical analyses, including the stimuli vectors of “HRV-food associated sound” (i.e. sound related to meat treats, for half of the dogs it was the high-pitched tone) and “LRV-food associated sound” (i.e. sound related to no-sugar fibre cookies, for half of the dogs it was the high pitched tone). Regressors were convolved with the canonical hemodynamic response function modelled as a Gamma function. Each run was analysed individually in a first level, on each run we contrasted each tone versus silence. With the resulting individual statistical parametric maps, at the group level, we performed a random-effects analysis involving data from all twenty participants. Region-of-interest (ROI) analyses were performed in two bilateral anatomically defined ROIs (i.e. the CN and amygdala). For each ROI, the average per cent BOLD signal change was extracted for the individual contrast images and then analysed with a 2 × 2 × 2 mixed model ANOVA with repeated measures (hemisphere –right, left; training –pre-training, post-training; sound – “HRV-food associated sound”, “LRV-food associated sound”). Data is provided as Supplementary Material. To ensure that the Inter-scan interval (ISI, i.e. the number of days between the end of the behavioural experiment and the post-training session) did not influence the brain response during the post-training runs we ran a supplementary GLM analysis. To assess the brain response to both sounds, we ran a whole-brain GLM analysis using only the two post-training runs and both sounds as regressors (cluster corrected, z > 2.3, p_cluster_ < 0.05). Then, we added the ISI as a covariate in the whole-brain GLM analysis by entering the demeaned ISI values as an orthogonalized explanatory variable concerning the brain response to sounds. Additionally, we analyzed the effect of including the ISI as a covariate on the response of the CN and the amygdala to both sounds in the post-training runs.

##### Representational similarity analysis

To assess if the representation of the stimuli in a given brain region changed in relationship with the training, we calculated the difference in dissimilarity between the stimuli representations before and after the training and correlated the difference with the behavioural change after training. We first created a dissimilarity map for “HRV-food associated sound” and “LRV-food associated sound” using a searchlight approach, this is, using the beta map for each sound before and after training, for a given voxel, we created a sphere (r = 3 voxels) and selected all the voxels included in the sphere. We calculated the dissimilarity as the correlation distance (1–Pearson correlation) between the response of the voxels. We assigned the dissimilarity to the centre and repeated the same process for all the voxels within the brain, thus creating a dissimilarity map. Using this procedure, we created two dissimilarity maps, one pre-training and one post-training. We calculated a dissimilarity change map by subtracting the pre-training from the post-training map. We created a dissimilarity change map for every dog by repeating this procedure.

To calculate a correlation map, we assessed the correlation between the difference in opening latencies for each dog and the dissimilarity change map for each voxel; we repeated this process for each voxel and each dog. To assess the correlation expected by chance, we followed a permutation-based approach similar to the one suggested by Stelzer, Chen and Turner^35^. This is, we randomly swapped the labels corresponding to the stimuli type before the analysis and repeated the procedure described above 100,000 times. Thus, creating a set of 100,000 correlation maps, we used these maps to assess the probability of a given correlation on each voxel. We filtered out all the voxels with a p > 0.001, creating a set of thresholded maps. Using the resulting maps, we assessed the cluster size distribution expected by chance. Using this distribution, we filtered out all the clusters with a p _cluster_ < 0.05.

### Results

#### Behaviour results

**Figure 4:**
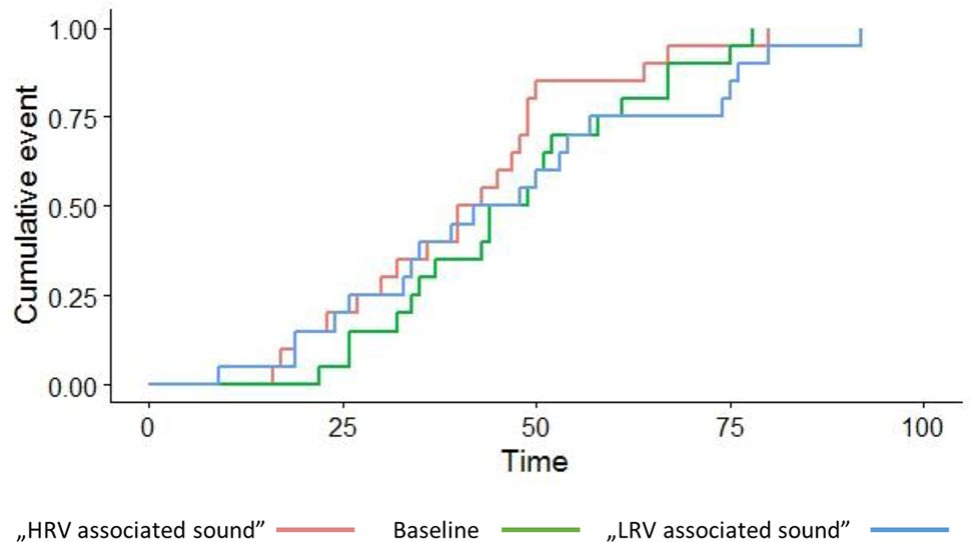
Cox mixed effect of parcel opening latencies (sec) in the three conditions (n = 20) – Experiment 2.

**Figure 5:**
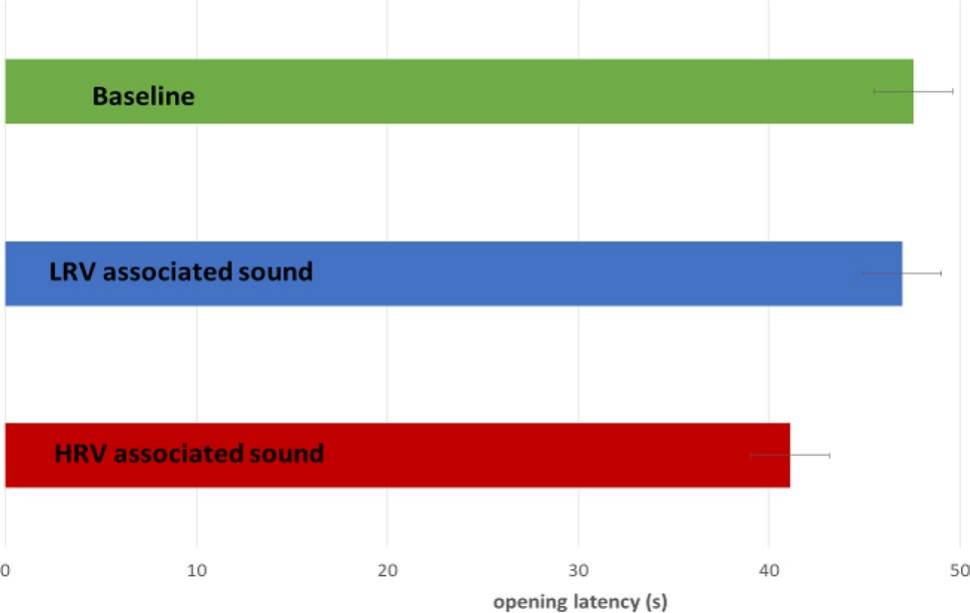
Group mean (± SE) of parcel opening latencies in the three conditions (n = 20) in Experiment 2. Means and ranges (sec): Baseline: 47.55 (22–75); LRV condition: 46.95 (9–92); HRV condition: 41.0 (16–80).

On the group level, opening latencies when hearing the HRV-food-associated sound did not differ significantly from baseline (exp (coef) = 0.668; p = *0.065*) or LRV-food associated sound (exp (coef) = 0.654; p = *0.063*) latencies (Fig. 4), while a similar trend to findings of the Experiment 1 can be found (Fig. 5).

Since in Experiment 1, we found parcel opening latencies upon hearing the sound associated with a higher reward value to be significantly lower than opening latencies upon hearing the sound associated with the lower reward value, we have compared parcel opening performances in the two experiments (Experiment 1 and 2) using another Mixed Effects Cox Regression Model and computing estimated marginal means. We have found a significant experimental effect in both the baseline (ß ± SE = − 1.17 ± 0.56, p = 0.038) and the lower reward value associated sound (ß ± SE = − 1.19 ± 0.55, p = 0.031) condition, while no experiment effect was found in the higher reward value associated sound condition (ß ± SE = − 0.71 ± 0.55, p = 0.194). Experiment 1 subjects were significantly slower in parcel opening than Experiment 2 subjects in both baseline and lower reward value-associated sound conditions. As no such difference was found in the higher reward value associated sound condition, the difference between the conditions was more pronounced in Experiment 1 than in Experiment 2.

#### fMRI results

**Figure 6:**
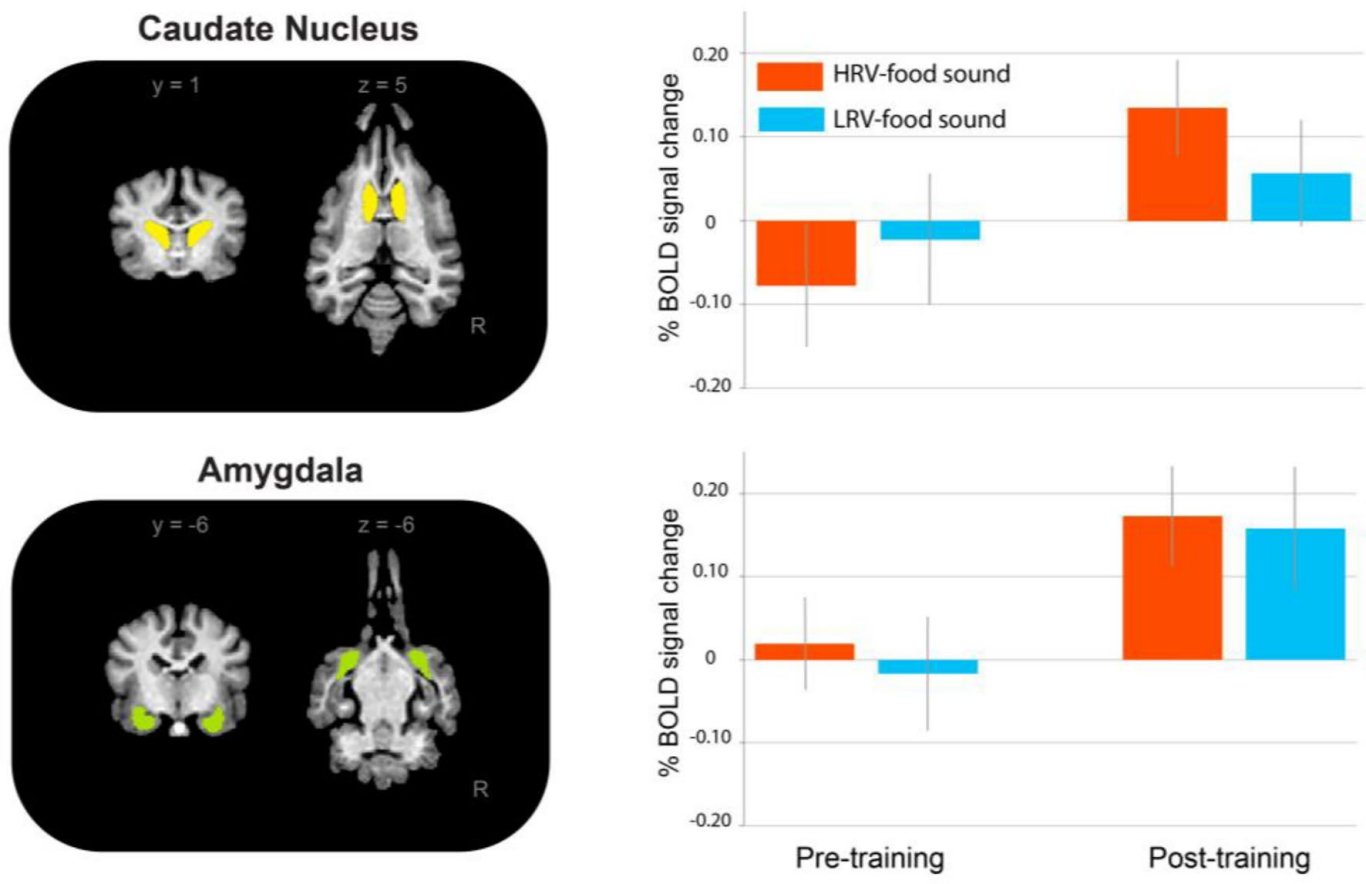
Mean percentage (± SE) of BOLD signal change by ROI for pre-training and post-training (n = 20).

As the Hemisphere main factor was not significant in any of the regions of interest, we removed it as a factor for the mixed model. The region of interest (ROI) analysis was performed using the average per cent BOLD signal change in each bilateral ROI. In the caudate nucleus (CN) we found a significant effect of the main factor of Training and a significant interaction between Training x Sound. In the amygdala, we found a significant effect of Training. Neither other factor’s effect nor their interactions were significant (Table 1, Fig. 6).

**Table 1:** Mixed model results (n = 20, df = 38). Significant results are marked by *.

Regions of interest (bilateral)	Fixed effects	F	p
Caudate nucleus	Training	4.097	0.049*
	Sound	0.157	0.694
	Training × sound	4.796	0.034*
Amygdala	Training	4.577	0.039*
	Sound	0.669	0.418
	Training × sound	0.153	0.698

Due to the significant interaction between Training × Sound in the CN, we performed planned comparisons of the CN response for both sounds in pre-and post-training. There were no significant differences in the CN response to the two different sounds in the pre-training “HRV-food associated sound”: M = − 0.08, SD = 0.33, range 0.4 to − 0.93; “LRV-food associated sound”: M = − 0.02, SD = 0.35, range 0.48 to − 0.69; t (19) = 0.82, p = 0.21), while we found significant differences in the CN response to the two different sounds in the post-training (“HRV-food associated sound”: M = 0.13, SD = 0.26, range 0.36 to − 0.3; “LRV-food associated sound”: M = − 0.06, SD = 0.28, range 0.44 to − 0.51; t (19) = − 1.96, p = 0.032).

The whole-brain GLM analysis of the two post-training runs, revealed a cluster in the left hemisphere with the peak z-value in the prorean gyrus (coordinates x = − 15, y = 3, z = 4) and extending to the CN, piriform cortex, insular cortex, rostral composite gyrus, and rostral ectosylvian gyrus for the contrast Sounds > Silence. Our analysis found no effect of the Inter-scan interval (ISI) as a covariate in the model. Additionally, the results showed no effect of the ISI in the amygdala response to both sounds. Although the CN showed a stronger response to both sounds when the model included the ISI as a covariate, a *t* test found no significant differences in the CN response between the models with and without the ISI as a covariate (Model without ISI as a covariate: M = 0.09, SD = 0.27, range − 0.47 to 0.58; Model with ISI as a covariate: M = 0.18, SD = 0.28, range − 0.47 to 1.17; t (19) = − 1.8, p = 0.078).

**Figure 7:**
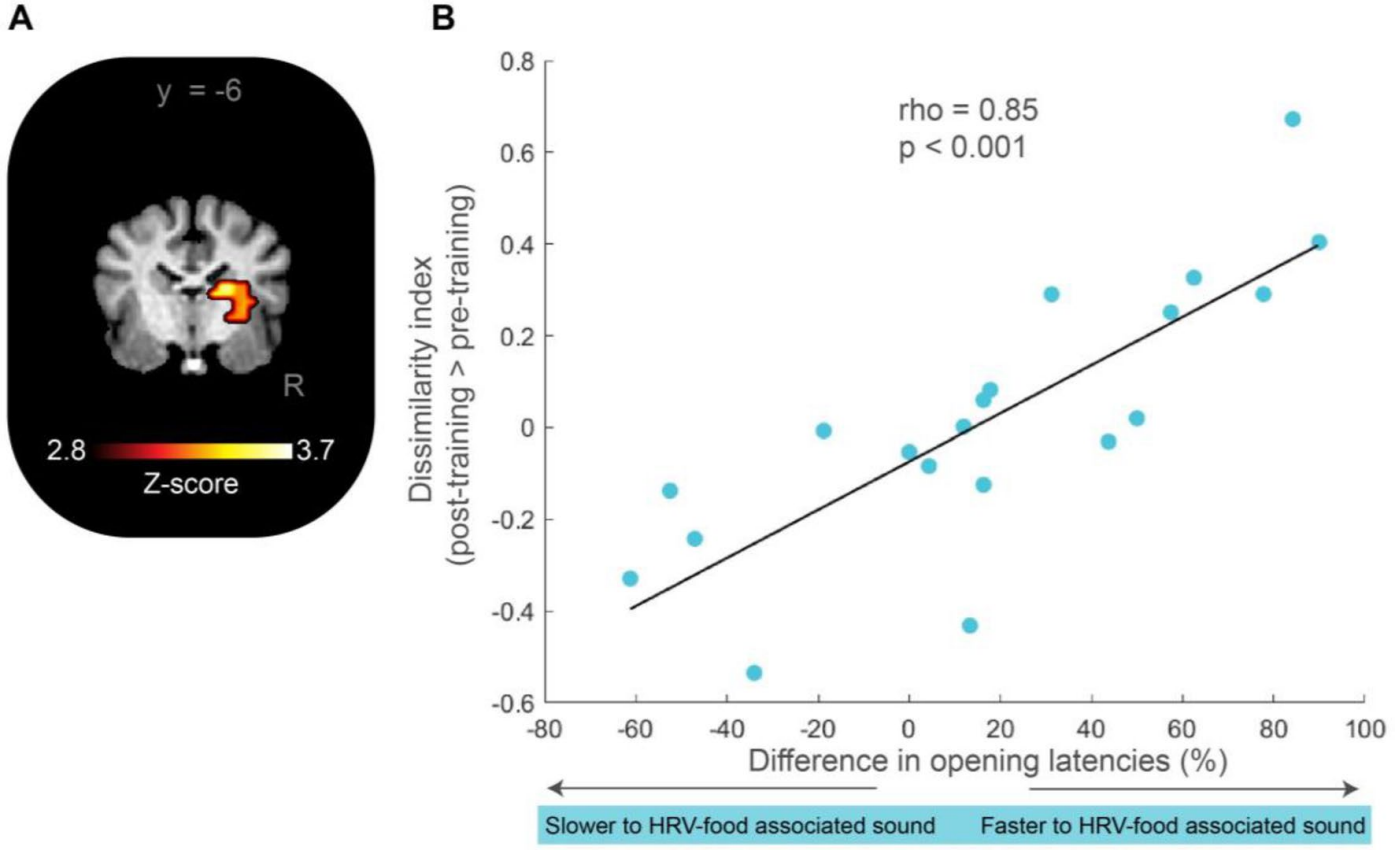
Representation of both sounds in the right CN was different after training and correlated with behavioural performance (n = 20). (**A**) Coronal view showing the resulting cluster of representational similarity analysis where the cerebral patterns to both sounds were statistically different post-training in comparison with pre-training (p < 0.001 p_cluster_ < 0.05). (**B**) Correlation between dissimilarity index in the right CN (sphere r = 3 voxels) and the difference in opening latencies. The empty circles represent dogs who did not open faster the parcel related to the better sound.

Finally, using a representational similarity analysis in the whole brain, we found a cluster in the right CN (32 voxels, p < 0.001 p_cluster_ < 0.05; coordinates: x = 9; y = − 5; z = 8) where the cerebral patterns to both sounds were different in the post-training in comparison with the pre-training. Moreover, the dissimilarity of cerebral patterns in the right CN (i.e. how different the two sounds are represented, a high value means that the representation between the sounds was more different post-training) showed a positive correlation with the performance in the behavioural task (r_s_ = 0.85, p < 0.001, Fig. 7).

## Discussion

Behavioural and cerebral responses of family dogs to two types of qualitatively different appetitive stimuli were investigated. Classical conditioning was used to teach dogs an association of two different tones with two types of food differing in reward value respectively. In Experiment 1, we designed a behavioural task to test if associative learning leads to behavioural changes mediated by motivation levels related to the expectation of rewards. Dogs opened a parcel faster when hearing the tone associated with a higher reward value in comparison with the tone associated with a lower reward value, suggesting a higher motivational state in connection with the expectation of a higher value reward. This finding is in line with those of Riemer and colleagues^36^, who also showed that reward quality affects dogs’ performance, probably mediated by the motivational state.

In Experiment 2, we repeated the training (with a slight increase in training trials) and the behaviour testing of Experiment 1 involving subjects specially trained to participate in fMRI experiments, to be able to compare findings of the behaviour testing with the measurement of brain response through fMRI. In this case, the difference in parcel opening latencies was not significant (p = 0.06) in the behavioural experiments, but a strong trend for lower latencies hearing the higher value food-associated sound could be observed. Based on owner reports and our observations during the first training session, we find the possibility that some fMRI-trained subjects did not have a clear preference for cooked ham over unsweetened fibre cookies unlikely. A possible explanation of slightly weaker differences in opening latencies on the group level could be that dogs participating in Experiment 2 were pre-selected by the fact that they were extensively trained for the fMRI, and this could have resulted in these subjects being more inclined to perform a task with proficiency, regardless of the expected reward value ^37^. Indeed, when comparing opening latencies in the two experiments, the results show that participants in Experiment 2 were faster in baseline and lower reward value food conditions than participants in Experiment 1, while no difference was found in the high reward value food condition. This supports the hypothesis that fMRI-trained dogs in Experiment 2 were overall more motivated to solve the task in all conditions independently from reward quality, thus the difference between HRV and LRV conditions became less pronounced. This may explain why the behavioural manifestation of association formation is not as evident in Experiment 2 (only trend) as it was in Experiment 1 (significant differences). The dogs trained for fMRI underwent successful training to remain still during MRI scans, which typically requires several months of weekly training^38^. Based on our experience and the STRANGE framework^37^, we suggest that the group of fMRI-trained dogs may differ from the general population of dogs in terms of their social background (i.e. exposure to more social learning opportunities), trappability and self-selection (i.e. individuals with specific characteristics being more likely to participate, interestingly this bias is applied to both, the dog and their caregiver), rearing history (i.e. exposure to more enrichment and social stimulation), acclimation and habituation (i.e. more prior visits to the Department of Ethology where the behaviour tests were conducted), and experience (i.e. a longer history of experimentation). Currently, a sampling bias in fMRI-trained dogs seems inevitable for conducting fMRI experiments without compromising the well-being of the dogs. Therefore, we suggest that the behaviour data from Experiment 1 could be more generalizable.

Regarding the cerebral results, the amygdala response increased for both sounds post-training, supporting the role of the amygdala in learning as we trained subjects to associate both sounds with an appetitive stimulus^5,12,22^. However, the amygdala response was similar to both sounds, suggesting that the amygdala was not involved in the discrimination of the two rewards of different qualities^14,15^. Interestingly, these results align with a previous study that demonstrated a bigger response in the amygdala to a stimulus associated with a reward compared to stimuli associated with nothing^22^. Our results suggest that the amygdala may have a broad response to positive learning rather than a sensitivity to the hedonic value of each reward. The CN response also increased post-training for both sounds, however in this case the post-training response was affected by the sound. Post-training, the CN response was greater to the higher-quality food sound compared to the lower-quality food sound, which suggests that the training led to the establishment of two specific representations. This result corroborates earlier findings suggesting the importance of the CN in reward representation in dogs related to food representation(e.g.^16–18^). Besides, CN is also related to the integration of lexical and intonational information in praise words^23^.

The representational similarity analysis results in the whole brain showed that the cerebral patterns in the CN representing the two sounds are different post-training, suggesting a key role of the CN in reward representation. Thus, our results show a qualitative reward representation in the CN and not only processing of the presence/absence of reward. A critical role of the CN in reward evaluation has been reported also in primates^39^. Similar results have been found on different quality rewards associated with conditioned stimuli in the visual modality^40^. In humans, besides the orbitofrontal cortex^41^ and the ventral striatum^42,43^, the CN has also been shown to process reward-quality information^44^.

In this study, we found a link between the performance in the behavioural task and the post-training neural representation of the two different sounds in the right CN. Specifically, the dissimilarity of cerebral patterns showed a positive correlation with the performance in the behavioural task. This means, that participants who opened the parcel faster upon hearing the sound associated with the HRV-food, showed more pronounced post-training differences in the representation patterns in the CN of the two sounds. A recent study showed that the CN response to a visual signal related to a reward predicts the success of training service dogs^45^. The authors suggested that a more pronounced response in the CN may indicate a higher motivational state. Our results also suggest a higher motivational state, as we only found this relationship during post-training. One potential limitation of the fMRI analysis was the use of a human hemodynamic response function (HRF). A recent methodological visual study indicated that using a tailored dog HRF can enhance fMRI detection power^46^. Future research will determine whether auditory studies with sparse sampling can similarly benefit from a tailored dog HRF.

In summary, dogs could associate different sounds with appetitive stimuli of different qualities. This learning led to a higher motivation to solve a task when hearing their “HRV-food associated sound”. At the neural level, the training led to an increase in amygdala activation towards both sounds. In contrast, higher activation in the CN was detected related to the “HRV-food associated sound” post-training. Besides having a distinguishable response to the two qualitatively different appetitive stimuli, a relationship between the representation of those and the behaviour has been shown in the right CN. We suggest the CN as the candidate to integrate the motivation to solve the behavioural task (measured as latencies) and the reward representation due to associative learning, as we found a direct relationship between behaviour and cerebral response in this region.

### Ethical statement

The reported research project is in accordance with the Ethical Guidelines of Research in Hungary, the behaviour experiment has been conducted with the permission of the National Animal Experimentation Ethics Committee (NÉBIH Állatkísérleti Tudományos Etikai Tanács) (number of ethical permission: PE/EA/1505-5/2017). The fMRI experiment was conducted at the Department of Neuroradiology, Medical Imaging Centre, Semmelweis University, Budapest, also with the permission of the National Animal Experimentation Ethics Committee (NÉBIH Állatkísérleti Tudományos Etikai Tanács) (number of ethical permission: KA-1719/PEI/001/1490-4/2015). Owners volunteered with their dogs to participate in the study, did not get any monetary compensation and gave written consent. All owners signed an informed consent form. Dogs and owners could freely decide to leave the sessions at any time. Methods are also in agreement with ASAB guidelines.

### ARRIVE guidelines

Authors state that this study is reported in accordance with ARRIVE guidelines. All procedures were performed in accordance with relevant guidelines.

### Supplementary Information

Supplementary Information.

## Data Availability

All data generated or analysed during this study included in this published article are available in Zenodo at https://doi.org/10.5281/zenodo.7626042 and its Supplementary Information files: SupplementaryMaterial_Data.pdf.
