# Supplementary material for: Representation of rewards differing in their hedonic valence in the caudate nucleus correlates with the performance in a problem-solving task in dogs (Canis familiaris)
Source: Sci Rep. 2023 Sep 1;13:14353. doi: 10.1038/s41598-023-40539-1 (PMC10474021; doi:10.1038/s41598-023-40539-1)
Supplement: Supplementary file 1 — Supplementary Information. [file 41598_2023_40539_MOESM1_ESM.pdf]

Subject data

Experiment 1

	Name	Breed	Sex	Age in months
1	**Szotyi**	Chihuahua	neutered female	30
2	**Floyd**	Golden retriever	male	30
3	**Walter**	Golden retriever	neutered male	72
4	**Mignon**	Mixed	neutered female	30
5	**Poppy**	Jack Russel terrier	neutered female	12
6	**Zajec**	Airedale terrier	neutered female	46
7	**Fruska**	Hungarian Vizsla	male	72
8	**Magor**	Hungarian Vizsla	female	72
9	**Panka**	Hungarian Vizsla	neutered female	120
10	**Kócos**	Mixed	neutered male	72
11	**Doris**	Groenendael	neutered female	108
12	**Robi**	Hungarian Vizsla	neutered male	36
13	**Twister**	Boxer	neutered male	84
14	**Joker**	Parson Russel terrie	neutered male	42
15	**Luca**	Mixed	neutered female	78
16	**Szálka**	Mixed	neutered male	20
17	**Zebulon**	Golden retriever	neutered male	120
18	**Joey**	Australian shepherd	neutered male	62
19	**Trinity**	Jack Russel terrier	neutered female	65
20	**Clyde**	Springer spaniel	male	13

Experiment 2

	Name	Breed	Sex	Age in months
1	**Akira**	Labradoodle	neutered female	42
2	**Alma**	mixed breed	neutered female	108
3	**Bodza**	Golden retriever	neutered female	74
4	**Bran**	Border Collie	neutered female	90
5	**Demi**	White Swiss shephe	neutered female	66
6	**Döme**	Cocker spaniel	neutered male	70
7	**Kara**	mixed breed	neutered male	56
8	**Kósza**	Belgian shepherd	male	12
9	**Kunkun**	Border Collie	neutered male	73
10	**Maverick**	Border Collie	male	121
11	**Maya**	Golden retriever	neutered female	101
12	**Mirza**	Springer spaniel	female	21
13	**Mokka**	Border Collie	neutered female	23
14	**Monty**	Border Collie	neutered male	103
15	**Nara**	Cocker spaniel	neutered female	24
16	**Nia**	Border Collie	neutered female	102
17	**Odin**	Border Collie	neutered male	68
18	**Pán**	Australian shepherd	neutered male	47
19	**Pax**	Cocker spaniel	neutered female	23
20	**Sander**	Golden retriever	neutered male	78

Behaviour study

Experiment 1

	Name	Opening latency in seconds: HRV Food sound:	LRV Food sound:	Baseline:
1	**Szotyi**	27	48	50
2	**Floyd**	68	69	105
3	**Walter**	24	31	20
4	**Mignon**	93	145	158
5	**Poppy**	19	58	18
6	**Zajec**	14	10	36
7	**Fruska**	65	82	87
8	**Magor**	82	44	62
9	**Panka**	71	103	105
10	**Kócos**	96	67	157
11	**Doris**	26	57	61
12	**Robi**	27	59	32
13	**Twister**	39	102	65
14	**Joker**	47	61	70
15	**Luca**	71	47	52
16	**Szálka**	78	89	98
17	**Zebulon**	89	86	55
18	**Joey**	22	32	47
19	**Trinity**	28	76	47
20	**Clyde**	35	58	35

Experiment 2

	Name:	HRV Food sound:	LRV Food sound:	Baseline:
1	**Akira**	64	92	75
2	**Alma**	49	19	44
3	**Bodza**	80	80	52
4	**Bran**	45	53	44
5	**Demi**	23	24	22
6	**Döme**	36	54	78
7	**Kara**	48	39	49
8	**Kósza**	49	57	43
9	**Kunkun**	40	76	58
10	**Maverick**	27	48	61
11	**Maya**	43	50	67
12	**Mirza**	32	42	35
13	**Mokka**	40	19	51
14	**Monty**	67	75	26
15	**Nara**	16	26	34
16	**Nia**	19	35	26
17	**Odin**	47	74	37
18	**Pán**	30	34	32
19	**Pax**	50	33	67
20	**Sander**	17	9	50

fMRI study - ROIs

	Pre-training		**Caudate Nucleus**	Post-training	
Name	HRV-food sound	LRV-food sound		HRV-food sound	LRV-food sound
Akira	0,40	0,48		-0,09	-0,10
Alma	0,00	-0,20		0,09	-0,11
Bodza	0,02	0,17		-0,24	-0,18
Bran	-0,11	-0,58		0,57	0,58
Demi	0,29	0,30		-0,08	-0,51
Döme	0,08	0,37		0,31	0,29
Kara	0,00	-0,02		0,05	0,14
Kósza	-0,06	0,02		-0,19	-0,18
Kunkun	-0,56	0,17		0,14	0,09
Maveric	-0,18	0,41		-0,13	-0,22
Maya	-0,03	-0,43		0,34	0,52
Mirza	0,03	0,07		0,59	0,44
Mokka	0,40	0,17		-0,01	0,08
Monty	0,21	0,30		0,28	0,00
Nara	-0,93	-0,62		-0,30	-0,20
Nia	0,25	0,13		0,31	0,35
Odín	-0,35	-0,02		0,20	0,25
Pán	-0,49	-0,69		0,36	0,09
Pax	-0,27	-0,25		0,13	-0,15
Sander	-0,25	-0,24		0,33	-0,07

	Pre-training		**Amygdala**	Post-training	
Name	HRV-food sound	LRV-food sound		HRV-food sound	LRV-food sound
Akira	0,45	0,24		0,23	0,42
Alma	0,38	0,22		-0,17	-0,57
Bodza	0,08	-0,27		0,26	0,19
Bran	-0,10	-0,02		0,38	-0,16
Demi	-0,53	-0,70		0,39	0,51
Döme	0,49	0,64		0,17	0,06
Kara	-0,03	-0,01		0,15	0,33
Kósza	-0,06	-0,31		0,40	0,28
Kunkun	-0,03	0,20		0,40	0,47
Maveric	0,12	0,09		-0,03	-0,27
Maya	-0,13	-0,11		-0,05	0,49
Mirza	-0,24	0,00		0,23	0,15
Mokka	0,29	-0,18		0,89	0,85
Monty	-0,01	0,20		-0,16	-0,12
Nara	-0,04	0,05		-0,42	-0,35
Nia	0,15	0,00		0,24	0,24
Odín	-0,14	0,29		0,17	0,34
Pán	0,00	-0,09		0,02	0,21
Pax	0,05	-0,02		0,16	0,12
Sander	-0,33	-0,62		0,19	-0,05
